# Lasting effects of early-life oxytocin treatment on LTP and episodic memory in a mouse model of Fragile X syndrome

**DOI:** 10.1038/s41386-026-02423-0

**Published:** 2026-04-28

**Authors:** Jasmine Chavez, Aliza A. Le, Julie C. Lauterborn, Brittney M. Cox, Yousheng Jia, Gary Lynch, Christine M. Gall

**Affiliations:** 1 Department of Anatomy and Neurobiology, University of California, Irvine, CA USA; 2 Department of Psychiatry and Human Behavior, University of California, Irvine, CA USA; 3 Department of Neurobiology and Behavior, University of California, Irvine, CA USA

**Keywords:** Long-term potentiation, Autism spectrum disorders, Hippocampus

## Abstract

Deficits in episodic memory are a debilitating feature of Fragile X syndrome (FXS) and other congenital autism spectrum disorders (ASDs). There is evidence that oxytocin (OXT) treatments can improve sociability in persons with ASD and related animal models, encouraging the idea that benefits might extend to cognitive function. We tested this possibility in male FXS model, *Fmr1*-knockout (KO) mice. Intranasal treatments with OXT or saline were given daily during the second or fifth postnatal week, and effects on social behavior, spatial and episodic memory, and hippocampal synaptic plasticity were assessed in adulthood. Saline-treated *Fmr1*-KOs exhibited profound deficits in social recognition, object location memory, what-when-where components of episodic memory and long-term potentiation (LTP) in both the CA3-CA1 and lateral perforant path (LPP) systems; NMDAR-mediated components of LPP responses were also impaired. OXT treatments during the second week postnatal normalized all of these functions in *Fmr1*-KOs assessed in adulthood; this included restoration of initial stages of CA3-CA1 LTP and granule cell NMDAR-mediated currents. In hippocampal slices from naïve adult male *Fmr1*-KO mice, bath-applied OXT treatment restored LTP in CA1 but not the LPP, indicating pathway-specific effects. Intranasal OXT treatments during the 5^th^ week postnatal did not have enduring effects in either genotype. The present evidence that early OXT treatment corrects a broad range of cognitive and synaptic plasticity deficits in *Fmr1*-KO mice identifies a clinically plausible strategy for normalizing hippocampal function in ASD and FXS, and highlights the presence of a critical developmental window for effective intervention.

## Introduction

Fragile X syndrome (FXS) is the most common inherited form of intellectual disability; it is approximately two-fold more prevalent in males than females [1, 2] and has high comorbidity for autism spectrum disorder (ASD) [3]. FXS arises from hypermethylation and silencing of the *Fmr1* gene [4–6], which encodes fragile X messenger ribonucleoprotein 1 (FMRP), an mRNA-binding protein that influences synaptic function at pre- and post-synaptic sites [7, 8]. Congenital absence of FMRP gives rise to learning impairments as well as sociability deficits, sensory hyperexcitability, seizures, and anxiety [9–11]. Pharmacological interventions for FXS individuals have focused on symptomatic treatment of behavioral and psychiatric disturbances (e.g., anxiety, aggression) [12, 13], but there has been little progress in developing treatments for cognitive disabilities that accompany the disorder.

Recent studies have investigated the possibility of using the hypothalamic neuropeptide oxytocin (OXT) to treat psychiatric disturbances associated with ASDs [14–16]. OXT plays an important role in social behavior [17, 18], and reduced OXT levels are observed in autistic individuals [14, 19, 20] and in animal models of ASD [21–23], including FXS model, *Fmr1*-knockout (KO) mice [24]. This suggests that early deficits in hypothalamic OXT expression may contribute to sociability issues with ASD. In support of this, Peñagarikano et al. found that intranasal OXT administration (iOXT) from postnatal day (P) 7 to P21 increased OXT levels in paraventricular hypothalamus (PVN) and normalized social recognition behavior in *Cntnap*2-KO mice [21]. Other work corroborated this basic finding [22, 25, 26], including evidence that iOXT given from P12 to P16 improved social recognition and novel object recognition in male *Fmr1*-KOs tested as adults [24]. Whether the benefits of early-life OXT treatment extend to impairments in episodic memory and its neurobiological underpinnings has not been examined.

Episodic memory, a form of incidental encoding that is critical for orderly thinking [27, 28], is impaired in ASDs [29–31]. It organizes the flow of everyday experiences into autobiographical units that include context-dependent information about the identities (“What”), locations (“Where”), and serial order (“When”) for a set of cues, and is accomplished without practice or overt rewards. Episodic memory, and the associated ability to encode temporal order and plan, is impaired in individuals with *Fmr1* mutations, as in ASD, whereas semantic memory is relatively spared [29, 32–37]. Acquisition and retrieval of episodic information depend on the hippocampus [38, 39], a structure that is disturbed in *Fmr1-*KOs. In particular, although baseline synaptic transmission seems normal, two distinct forms of memory-related long-term potentiation (LTP) are substantially impaired [40–43]; such defects in synaptic plasticity could underlie FXS-related episodic memory problems. The hippocampus expresses OXT receptors [44–47] and exhibits changes in neuronal activity with OXT treatment [48]. Accordingly, we tested the possibility that early OXT treatment might enhance hippocampal synaptic plasticity and episodic memory in adult *Fmr1*-KO mice. These initial studies focused on males due to the higher incidence of FXS and greater severity of cognitive impairments in males as compared to females carrying a full mutation [1, 2]. The results reveal enduring positive effects of early OXT treatment, indicating that such interventions can have long-lasting beneficial effects on cognitive function in FXS.

## Methods and materials

### Animals

Male FVB129 *Fmr1*-KO and FVB129 wild-type (WT) mice were bred in-house, weaned at P21 and group-housed (12-h light/dark cycle; food and water *ad libitum*) with same-sex littermates. Protocols were approved by the Institutional Animal Care and Use Committee (IACUC) and were consistent with the Guide for the Care and Use of Laboratory Animals.

### Intranasal oxytocin treatment

Oxytocin (1 µg/µL) or 0.9% NaCl (SAL) was administered intranasally (2 µL per nostril), daily from P7-P13 or P30-P36; thus, age-matched groups received equivalent handling. Intranasal OXT administration was used because, at a range of ages extending well into adulthood, it leads to greater and more enduring increases in brain OXT levels than are accomplished with peripheral administration, it increases OXT levels in hippocampus [47, 49, 50], and it elicits neuronal responses throughout the brain, including broadly distributed increases in c-fos expression in mice treated at 6 weeks of age [51]. Moreover, intranasal administration is favored in human tests for effects on social behavior [52–55]. Analytical procedures were initiated when mice reached 2–4 months of age.

### Behavioral assays

**Fig. 1:**
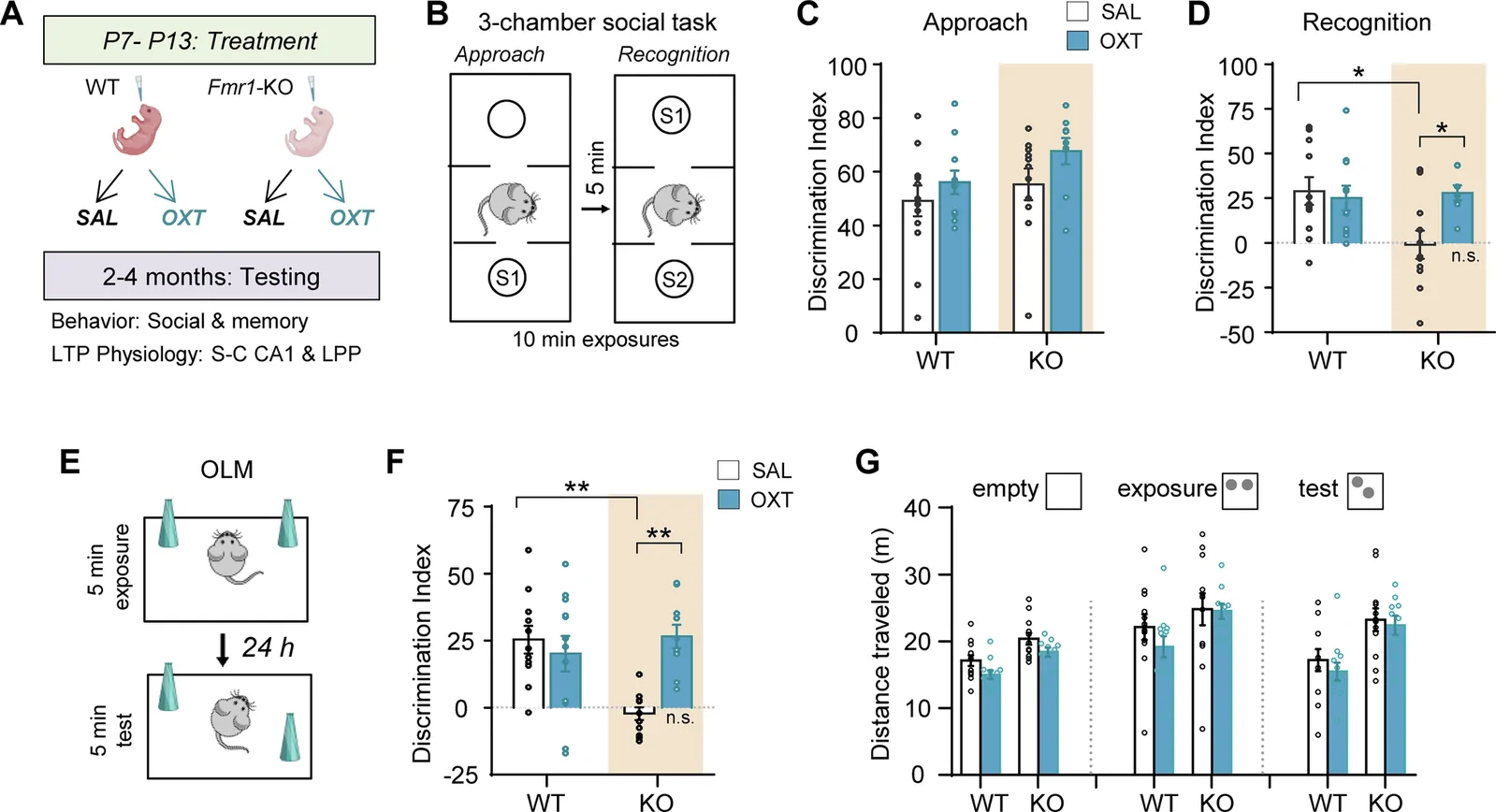
Early oxytocin (OXT) treatment improves social recognition and spatial memory in male *Fmr1*-KO mice. **A** Timelines for early OXT and saline (SAL) treatment (daily, postnatal day (P) 7 to P13) and testing (2-4 mo of age) for behavioral and electrophysiological effects of genotype (*Fmr1*-KO vs WT) and treatment. **B** The 3-chamber sociability task entailed two phases. *Approach*: Mice were exposed to an empty cup or a stranger mouse (S1, under the cup). *Recognition*: Mice explored the apparatus with side chambers containing a novel stranger mouse (S2) and S1. **C** In Approach, all groups preferentially explored the S1 mouse over the empty cup, exhibiting high discrimination index (DI) scores (*F*_(1, 40)_ = 0.255, *p* = 0.617, 2-way ANOVA (interaction). **D** In Recognition, both WT groups preferentially sampled the novel mouse without effect of OXT treatment; SAL-treated KOs did not differentially explore the novel S2 mouse (*F*_(1,37)_ = 5.149, *p* = 0.029; *p = 0.02 KO-SAL vs. WT-SAL), whereas OXT-treated KOs did (*p = 0.047 KO-OXT vs. KO-SAL) with behavior comparable to WTs (n.s. *p* = 0.99 KO-OXT vs. WT-OXT). **E** Object location memory (OLM) paradigm entailed exposure to two identical objects placed near adjacent corners. After 24 h, the mice were reintroduced to the chamber with one object relocated. **F** Both WT groups preferentially explored the novel-location object, whereas SAL-treated KOs did not (*F*_(1,40)_ = 11.6, *p* = 0.002; **p = 0.002 WT-SAL vs. KO-SAL). However, *Fmr1*-KOs given OXT preferentially explored the relocated object (**p = 0.002 KO-SAL vs. KO-OXT) with DIs comparable to WT groups (n.s. *p* = 0.799 KO-OXT vs. WT-OXT). **G** Distance traveled during the OLM habituation (empty chamber), exposure, and test sessions. There was a modest effect of genotype with KOs traveling more than WTs (empty chamber: *F*_(1,42)_ = 18.8, *p* < 0.0001, exposure: *F*_(1,42)_ = 4.64, *p* = 0.037, and test: *F*_(1,42)_ = 17.5, *p* = 0.0001), but post-hoc analysis did not detect an OXT influence on KO or WT measures in any trial. Statistics (**C**, **D**, **F**, **G**): two-way ANOVA (interaction) with Tukey post-hoc comparisons indicated as *p < 0.05 and **p < 0.01. See Table S2 for all statistical comparisons.

**Fig. 2:**
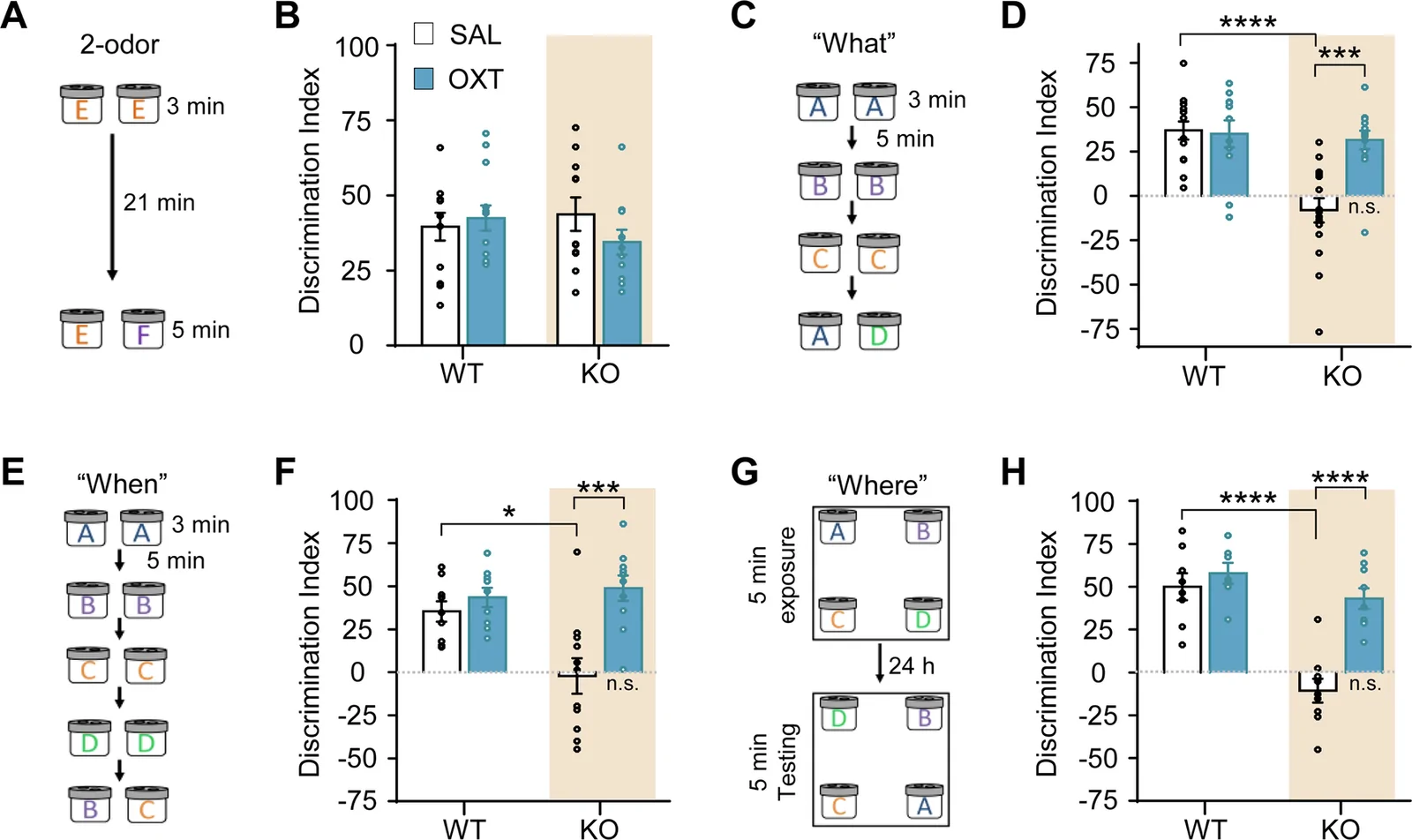
Episodic memory is restored in *Fmr1*-KOs by early OXT treatment. Behavioral tests evaluated odor discrimination and acquisition of 3 components of episodic memory ("What", "Where", "When") in adult male WT and *Fmr1*-KO mice given early (P7-P13) OXT or SAL treatments. In the 2-odor task (**A**), all groups preferentially discriminated novel odor F from familiar odor E (**B**; *F*_(1,43)_ = 1.72, *p* = 0.196). In the serial episodic "What" task (**C**), WTs ( ± OXT) and *Fmr1*-KOs given OXT preferentially explored novel odor “D” vs. familiar odor “A”, whereas SAL-treated *Fmr1*-KOs did not (**D**; *F*_(1,50)_ = 10.8, *p* = 0.002). **E**, **F** In the serial episodic "When" task (**E**), SAL-treated KOs did not discriminate the test odors, whereas KOs given early OXT treatment preferentially explored less recently sampled odor “B” with DI scores comparable to WTs (**F**; *F*_(1,35)_ = 7.34, *p* = 0.01). **G**, **H** In the episodic "Where" task (**G**), WT groups ( ± OXT) preferentially explored novel-location cues (A:D), whereas SAL-treated KOs did not. In contrast, *Fmr1*-KOs given early OXT treatment had high discrimination index scores comparable to WTs (*F*_(1,29)_ = 11.0, *p* = 0.003). Statistics for all sets: two-way ANOVA (interaction) with Tukey’s post-hoc comparisons indicated as: *p < 0.05, ***p < 0.001, ****p < 0.0001, and n.s. *p* > 0.05 for WT-OXT vs. KO-OXT. The legend in (**B**) also applies to (**D**, **F**, **H**). See Table S2 for all statistical comparisons.

Behavioral sessions, performed from 10AM to 3PM, included the three-chamber sociability task, object location memory, 2-odor discrimination and episodic memory tasks as described elsewhere [41, 56–59] and in Supplementary Materials, and illustrated in Figs. 1 and 2. Statistical analyses and raw sampling times are in Tables S2 and S3.

### Extracellular field and whole-cell current-clamp electrophysiology

Acute hippocampal slices were used for electrophysiological analysis of CA3-CA1 Schaffer-commissural (S-C) projections and lateral perforant path (LPP) input to the dentate gyrus (DG) (field responses, interface chamber, 31°C), and whole cell measures of glutamate receptor currents (submerged chamber) as described [42, 56, 60] (also Supplementary Materials). Experiments were initiated ≥ 1.5 hr after slice preparation. LTP was induced using theta burst stimulation (TBS; bursts of 4 pulses at 100 Hz, 200 ms between bursts) or high frequency stimulation (HFS; 1 sec/100 Hz) for S-C and LPP systems, respectively. LTP studies evaluated responses from at least 7 slices per group from ≥6 mice for the LPP and ≥4 mice for the S-C system. The N is the number of hippocampal slices for field recordings and the number of neurons for whole cell recordings.

### Hippocampal slice bath-treatments

Stock solutions of physostigmine (Tocris, 0622) and OXT (Cell Sciences, CRO300A) were diluted into artificial cerebrospinal fluid (aCSF) [56] on the day of use for final bath concentrations of 10 µM and 1 µM, respectively. After collecting 20 min baseline recordings, reagents were infused into the slice chamber by introduction into the aCSF infusion line using a syringe pump (6 mL/hr) for 45 (physostigmine) or 30 (OXT, vehicle) minutes.

### Statistical analysis

Significance was determined using GraphPad Prism v6.0 (San Diego, CA). Two-way ANOVA with Tukey post-hoc analyses were used for genotype and drug comparisons. Frequency facilitation and theta burst response analyses used repeated-measures two-way ANOVA. Distance traveled and sampling time analyses used a repeated-measures mixed ANOVA. Input/output curve analysis used linear regression of slope.

## Results

### Early-life OXT treatment rescues social recognition in adult Fmr1-KO mice

Oxytocin treatment during the second and third postnatal weeks is reported to normalize social recognition behavior in ASD models [21, 22, 61], including *Fmr1*-KO [24] mice. To reevaluate this fundamental finding, we first tested if iOXT, as compared to intranasal SAL (iSAL), treatment once daily from P7 to P13, rescues social recognition in adult *Fmr1*-KOs using the 3-chamber paradigm [62] (Fig. 1A, B). *Fmr1*-KO and WT mice exhibited comparable social approach regardless of treatment (Figs. 1C and S1A). In the social recognition trial, both WT groups ( ± OXT) preferentially explored the novel over the familiar mouse. This discrimination was absent in *Fmr1*-KOs given iSAL but was restored to WT levels by iOXT (Figs. 1D and S1B).

### Early OXT treatment restores spatial and episodic memory in Fmr1-KOs

*Fmr1*-KOs exhibit deficiencies in object location memory (OLM) [41, 63]. We tested whether early-iOXT restores this hippocampus-dependent [64, 65] form of spatial learning (Fig. 1E) in *Fmr1*-KOs tested as adults. Wild-types ( ± OXT) preferentially explored the displaced object at testing, whereas SAL-treated KOs did not (Fig. 1F). In contrast, *Fmr1*-KOs given iOXT preferentially sampled the displaced object with cue sampling times and discrimination indices (DIs) comparable to WTs (Figs. 1F and S1C). The distance traveled during OLM trials was somewhat greater in *Fmr1*-KOs vs WTs, and iOXT did not influence this measure (Fig. 1G).

We previously showed *Fmr1*-KOs have pronounced deficits in encoding cue identity (episodic “What”) [42]. Here, the analysis was extended to assess the effects of genotype on the acquisition of “What”, “Where” and “When” information, and the effects of early iOXT treatment. The tasks [58] employed odor cues, which are of inherent interest to macrosmatic animals, and did not include practice or reward. We first tested four groups (WTs and KOs, given iOXT or iSAL) in 2-odor discrimination (Fig. 2A). In this, and subsequent episodic memory tasks, the total cue sample time was comparable for all groups during initial cue exposure sessions (Fig. S2). At 2-odor task testing, all mice preferentially explored the novel odor, indicating that simple odor discrimination, interest in novelty and related memory were unaffected by genotype or iOXT (Figs. 2B and S3A). To assess episodic “What” acquisition, mice were exposed to a sequence of odor pairs (A-A, B-B, C-C; Table S1), followed by a test pair containing previously sampled odor A and novel odor D (Fig. 2C). Wild-type mice ( ± OXT) preferentially explored the novel odor, denoting learning. This discrimination was absent in *Fmr1*-KOs given early iSAL but was restored to WT levels in those given early OXT treatment (Figs. 2D and S3B).

To assess acquisition of cue presentation order (episodic “When”), mice were exposed to a sequence of four odor pairs (A-A, B-B, C-C, D-D), followed by a test pair including odors B and C (Fig. 2E). Wild-type mice ( ± OXT) discriminated the less recent odor (B), thereby demonstrating acquisition of cue order, whereas iSAL-treated KOs did not. Oxytocin treatment fully restored “When” acquisition in the KOs (Figs. 2F and S3C). To test acquisition of cue location (episodic “Where”), mice explored a chamber containing 4 different odors placed near the arena corners; at testing 24 h later, positions of two diagonally opposed odors were swapped (Fig. 2G). Both WT groups ( ± OXT) preferentially investigated the novel-location odors, but iSAL-treated KOs did not. However, *Fmr1*-KOs given early iOXT preferentially explored the relocated odors with DIs comparable to that of WTs (Figs. 2H and S3D).

Although there was no effect of genotype or treatment on initial cue sampling times for the episodic memory tasks, the distance traveled in initial cue exposure trials was greater in KO vs WT mice (in agreement with prior reports [63]). This measure was not influenced by iOXT for the episodic “Where” task, but for serial odor tasks (“When”, “What”), the distance traveled was reduced by iOXT in both genotypes (i.e., there was no significant interaction between genotype and treatment) (Fig. S4).

### Early-life OXT treatment rescues Fmr1-KO Schaffer-Commissural (S-C) LTP

**Fig. 3:**
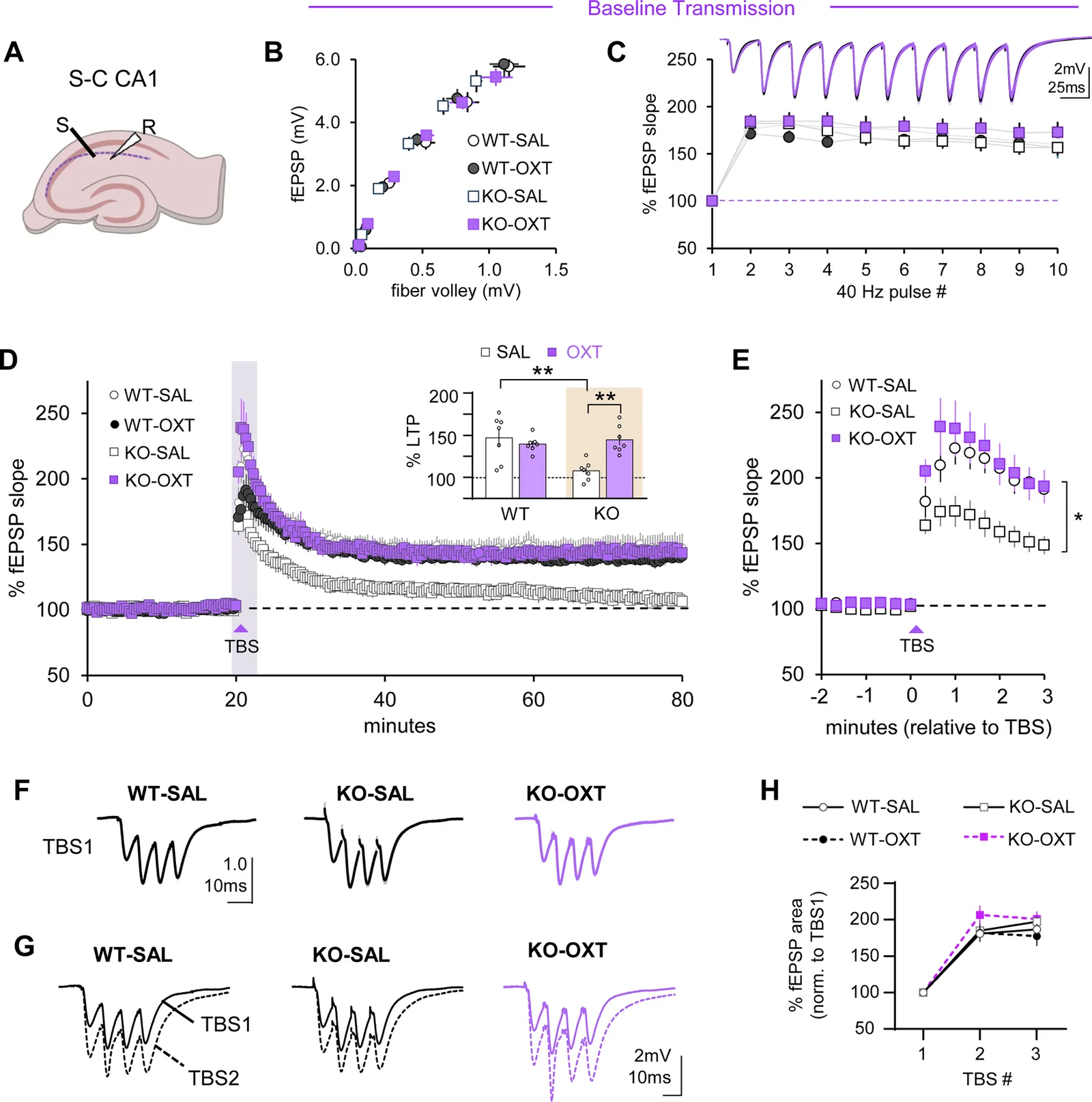
Postnatal OXT treatment restores Schaffer-commissure (S-C), field CA1 LTP. **A** Placement of stimulating (S) and recording (R) electrodes for analysis of S-C responses in hippocampal slices prepared from adult WT and *Fmr1*-KO mice treated with SAL or OXT (P7-P13). **B** Input (fiber volley amplitude)/output (fEPSP amplitude) curves were similar across groups with no effect of genotype or treatment (*F*_(3,190)_ = 1.87, *p* = 0.136; linear regression). **C** Responses to a ten-pulse, 40-Hz stimulation train were comparable across groups (*F*_(27,270)_ = 0.915, *p* = 0.59); legend as in (**B**). Superimposed traces (top) show mean ( ± SEM) fEPSP responses for WT-SAL (black) and KO-SAL (purple) mice. **D** Plot of fEPSP slopes shows that application of 3 theta bursts (theta burst stimulation, TBS), elicited stable S-C LTP in WT slices ( ± OXT) but not in slices from SAL-treated KOs. In contrast, in OXT-treated *Fmr1*-KOs, TBS elicited stable S-C LTP comparable to that in WTs. *Inset bar graph:* Mean LTP values from 55-60 min post-TBS (*F*_(1,24)_ = 11.04, *p* = 0.0028, 2-way ANOVA; **p < 0.01 post-hoc Tukey). **E** Expansion of the x-axis (from shaded area of panel **D**) shows that initial increases in S-C fEPSP size after TBS are smaller in SAL-treated KOs vs SAL-treated WTs, but are restored to WT levels in OXT-treated KOs (*F*_(30,270)_ = 4.142, *p* < 0.0001; post-hoc Tukey: *p < 0.05 for WT-SAL and KO-OXT vs. KO-SAL). **F** Traces show mean ( ± SEM) responses to the first theta burst (TBS1), normalized to the amplitude of the response to the first pulse (n = 7/group; note, error bars are hidden by data points). **G** Representative superimposed fEPSP responses to the first burst (TBS1, solid line) and the subsequent burst (TBS2, dashed line), illustrating the response facilitation from TBS1 to TBS2. **H** Facilitation in fEPSP areas across the three bursts was comparable among groups (*F*_(6,48)_ = 1.467, *p* = 0.2095); plots show mean (± SEM) values. Statistics (**C**, **E**, **H**): 2-way RM ANOVA (interaction) with post-hoc Tukey analyses.

Acquisition of spatial and episodic memory depends on hippocampus [39, 66, 67], and adult male *Fmr1*-KOs exhibit LTP deficits in two links in the prime hippocampal circuit, LPP innervation of the DG [42, 68] and S-C projections to CA1 [43, 69], that are implicated in episodic encoding [58] and OLM [64], respectively. Thus, early iOXT may rescue memory in *Fmr1*-KOs by restoring plasticity in these systems. We tested the effects of genotype and treatment on basal synaptic transmission and LTP in adult hippocampal slices. Recording electrodes placed in CA1 stratum radiatum measured population-spike free fEPSPs evoked by S-C afferents (Fig. 3A). S-C input/output curves did not differ between KOs and WTs (Fig. 3B), nor did frequency facilitation with gamma frequency (40 Hz) stimulation (Fig. 3C). Thus, the mutation does not appear to alter basal transmission or facilitated release produced by repetitive activation.

Prior work revealed S-C LTP deficits in *Fmr1*-KOs using near-threshold inducing stimulation [43, 69]. Thus, we evaluated LTP using a minimum induction protocol involving a train of three theta bursts; this induced stable CA1 LTP in slices from WTs but not *Fmr1*-KOs. OXT given from P7-P13 fully restored S-C LTP in adult *Fmr1*-KOs without influencing potentiation in WTs (Figs. 3D and S5A). This first use of the three-burst paradigm led to the unexpected observation that in WT mice, the response magnitude steadily increases across the first three stimulation pulses delivered after TBS. There was an equivalent increase in the slope of the first post-TBS response in SAL-treated WTs and KOs (181.9 ± 11.9 vs. 163.7 ± 6.3%, respectively; *p* = 0.201, Tukey's post-hoc), but subsequent increases in each pulse response were smaller in KOs vs. WTs (*p* < 0.025, post-hoc). Early-life iOXT normalized this post-induction facilitation in *Fmr1*-KOs assessed in adulthood (Fig. 3E). Thus, the mutation disrupts events occurring shortly after S-C LTP induction and this abnormality is stably corrected by early OXT treatment.

We tested for perturbations to theta-burst responses used to induce S-C LTP. Waveforms for each fEPSP in the initial TBS burst were expressed as a percent of the first pulse fEPSP amplitude; the mean burst response for each slice (Fig. 3F) was used to calculate the mean first burst area ( ± SEM) for all slices in three groups: WT + SAL (41.56 ± 0.85 msec*mV), *Fmr1*-KO + SAL (41.15 ± 0.90), and *Fmr1*-KO + OXT (39.64 ± 2.18). First burst responses were comparable across groups (One-way ANOVA (interaction): *F*_(2,19)_ = 0.5129, *p* = 0.607) and all exhibited a pronounced increase in response area between the first and subsequent bursts in a train (Fig. 3G). The three consecutive bursts elicited comparable fEPSP areas across treatments and genotypes (Fig. 3H). The within-train increase is largely due to reduced shunting inhibition and the addition of NMDAR-gated potentials to the fEPSPs [70–72]. These results show that *Fmr1* knockout does not measurably change synaptic responses evoked by individual theta bursts; thus, it leaves LTP induction steps intact but truncates the initial development of LTP expression. Early postnatal OXT treatment normalized the rapid response growth after TBS and fully restored S-C LTP in *Fmr1*-KOs without effect in WTs.

### Early-life OXT treatment restores LPP-LTP in adult Fmr1-KOs

**Fig. 4:**
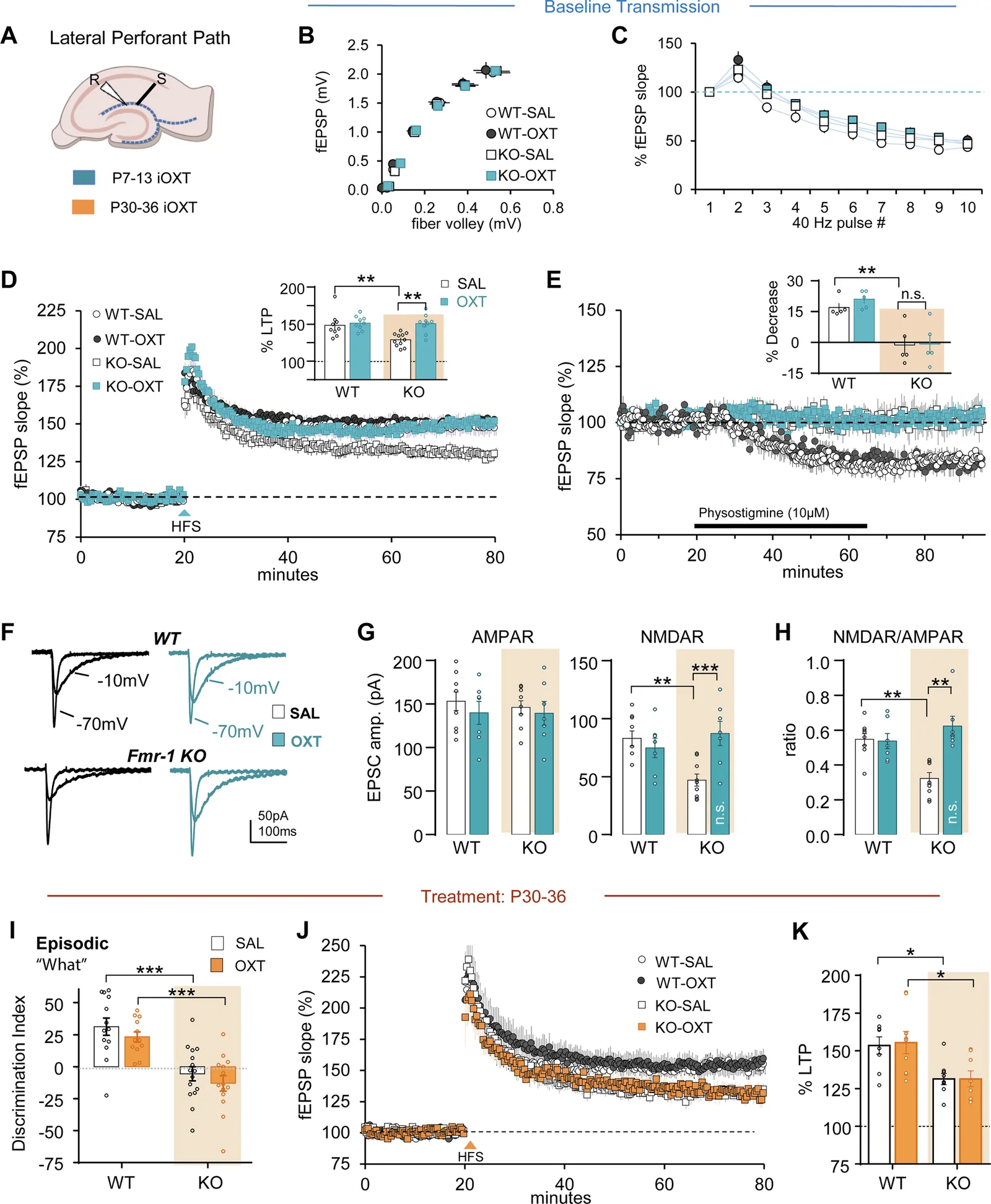
Early-postnatal, but not adolescent, OXT treatment rescues lateral perforant path (LPP) LTP in male *Fmr1*-KO mice. **A** Placement of stimulation (S) and recording (R) electrodes for studies of LPP (dashed blue line) input to the dentate gyrus in slices from WT and *Fmr1*-KO mice given SAL or OXT from P7-13 (blue) or P30-36 (orange); panels B-H and I-K show data for each period, respectively. **B** Input/output curves were comparable across groups (*F*_(3,118)_ = 0.109, *p* = 0.955; linear regression). **C** LPP stimulation with a ten-pulse 40-Hz train elicited a similar fEPSP response profile across groups (*F*_(27,234)_ = 1.28, *p* = 0.171; two-way RM ANOVA; legend as in (**B**). **D** After 20 min of baseline recording, LPP-LTP was induced with a 1-sec 100 Hz train (HFS). Stable and comparable LPP-LTP was induced in both WT groups. LPP-LTP was impaired in *Fmr1*-KOs given SAL, but was restored to WT levels in KOs given OXT. *Inset bar graph:* Mean potentiation at 55-60 min post-HFS (*F*_(1,34)_ = 5.61, *p* = 0.024, 2-way ANOVA interaction). Legend in (**D**) also applies to (**E**–**H**). **E** Physostigmine infusion depressed LPP fEPSPs in WTs ( ± OXT), but had no effect on responses in *Fmr1*-KOs with or without early OXT treatment (treatment: *F*_(1,16)_ = 0.4295; *p* = 0.522). *Inset graph:* % decrease in fEPSP slope 70-75 min after infusion onset. **F**–**H** Granule cell whole-cell recordings were used to assess NMDAR/AMPAR current ratios with LPP stimulation in adult slices. **F** Representative EPSC traces with membrane potential held at –10mV and –70mV for both WT and KO cases given early OXT or SAL treatment. **G** AMPAR currents were similar across all groups (interaction: *F*_(1,28)_ = 0.086, *p* = 0.77), whereas NMDAR-mediated currents were markedly lower in KO-SAL vs. WT-SAL cases, but restored to WT levels in KOs given early OXT treatment (*F*_(1,28)_ = 10.3, *p* = 0.003). **H** The NMDAR/AMPAR evoked current ratio was comparable in WTs ± OXT, significantly lower in KO-SAL cases, and restored to WT levels in the KO-OXT group (interaction: *F*_(1,28)_ = 14.5, *p* = 0.0007). **I** With adolescent treatments, WT mice ( ± OXT) preferentially explored the novel odor in the episodic “What” task, whereas *Fmr1*-KOs given SAL or OXT exhibited comparable behavior and no preference (interaction: *F*_(1,50)_ = 0.003, *p* = 0.96; Genotype: *F*_(1,50)_ = 40.0, *p* < 0.0001; post-hoc Tukey: ***p <0.001 KO-SAL vs. WT-SAL and KO-OXT vs. WT-SAL; n.s. *p* = 0.78 KO-SAL vs. KO-OXT). **J** Robust LPP-LTP was induced in slices from WT mice ( ± adolescent OXT), whereas LPP-LTP was of lower magnitude in *Fmr1*-KOs independent of treatment. **K** Mean LPP-LTP at 55-60 min post-HFS (from J; interaction: *F*_(1,30)_ = 0.028, *p* = 0.87; Genotype: *F*_(1,30)_ = 16.6, *p* = 0.0003; post-hoc Tukey: *p <0.05 KO-SAL vs. WT-SAL and KO-OXT vs. WT-OXT, n.s. *p* > 0.999 KO-SAL vs. KO-OXT); see (**I**) for legend. For (**D**, **E**, **G**, **H**): **p < 0.01, ***p < 0.001; n.s. for *p* > 0.05 for WT-OXT vs. KO-OXT.

Evidence that LPP-LTP is impaired in *Fmr1*-KOs [42, 68, 73] is of particular interest because the LPP-DG connection is critical for the acquisition of all three major components of episodic memory [58]. We tested if early OXT treatments, which rescued episodic memory, also normalize LPP-LTP (Fig. 4A). LPP input/output curves were comparable and unaffected by prior OXT treatment in WTs and *Fmr1*-KOs (Fig. 4B). Gamma frequency stimulation caused an initial within-train facilitation of fEPSPs followed by a decline to below baseline, an unusual response profile that reflects release variables [74]. This profile was intact in all four groups (Fig. 4C), suggesting that signal processing at the adult LPP-DG synapse is unaffected by genotype or early OXT treatment. Nevertheless, LPP-LTP was smaller in SAL-treated KOs relative to SAL-treated WTs. This *Fmr1*-KO impairment in LPP-LTP was eliminated by early OXT treatments that had no effect on potentiation in WTs (Figs. 4D and S5B).

Prior work showed that deficits in male *Fmr1*-KO LPP-LTP reflect both reduced NMDAR-mediated synaptic responses [42, 68, 73] and insufficient endocannabinoid signaling needed to trigger the presynaptic changes that express LPP-LTP [75, 76]. We previously showed that physostigmine infusion elicits cannabinoid receptor type 1 (CB1R)-dependent depression of LPP baseline responses [75] and that attenuation of this effect in *Fmr1*-KOs reflects deficient endocannabinoid signaling [42]. To test if early iOXT restores this endocannabinoid transmission, physostigmine was applied to hippocampal slices from iOXT- or iSAL-treated mice. The LPP-response depression was present and comparable in both WT groups but was absent in *Fmr1*-KOs regardless of OXT treatment (Two-way ANOVA (genotype): *F*_(1,16)_ = 35.49, *p* < 0.0001; Fig. 4E). This indicates that endocannabinoid signaling deficits persist in male *Fmr1*-KOs and that iOXT likely rescues LPP-LTP through effects on NMDAR-mediated currents. Whole-cell recordings showed that AMPAR-gated LPP-EPSCs were comparable in adult WT and *Fmr1*-KO mice and unaffected by early OXT treatment (Fig. 4F, G). However, NMDAR-mediated currents were significantly smaller in iSAL-treated KO vs WT mice (Fig. 4G). Early iOXT brought evoked NMDAR-mediated LPP responses in *Fmr1*-KOs to WT levels and, consequently, normalized the NMDAR/AMPAR current ratio in the mutants (Fig. 4H). These results describe a specific, enduring modification produced by early OXT treatment that contributes to both physiological (LPP-LTP) and behavioral (episodic memory) outcomes.

### Effects of iOXT given during adolescence

The hypothesis that early OXT treatment normalizes aberrant developmental trajectories associated with FXS suggests that the benefits of treatment may depend on the age of OXT administration. To test this, mice were given iOXT or iSAL during adolescence (daily, P30-P36, Fig. S6A), and assayed as adults for 2-odor discrimination, episodic “What” acquisition and LPP-LTP. Mice in all four groups (WT ± OXT; KO ± OXT) had comparably high DIs for two-odor discrimination (Fig. S6B, C). Adolescent iOXT did not affect behavioral measures or LPP-LTP in the WTs. SAL-treated KOs had a severe deficit in episodic “What” acquisition, and this was unaffected by adolescent iOXT treatment (Figs. 4I and S6D). Similarly, adolescent iOXT failed to offset the LPP-LTP impairment assessed in adult *Fmr1*-KOs (Figs. 4J, K and S5C). Thus, in male *Fmr1*-KOs, P30-P36 OXT treatment had no effect on episodic memory or the synaptic plasticity needed for its encoding.

### Effects of acute OXT treatment on LTP

**Fig. 5:**
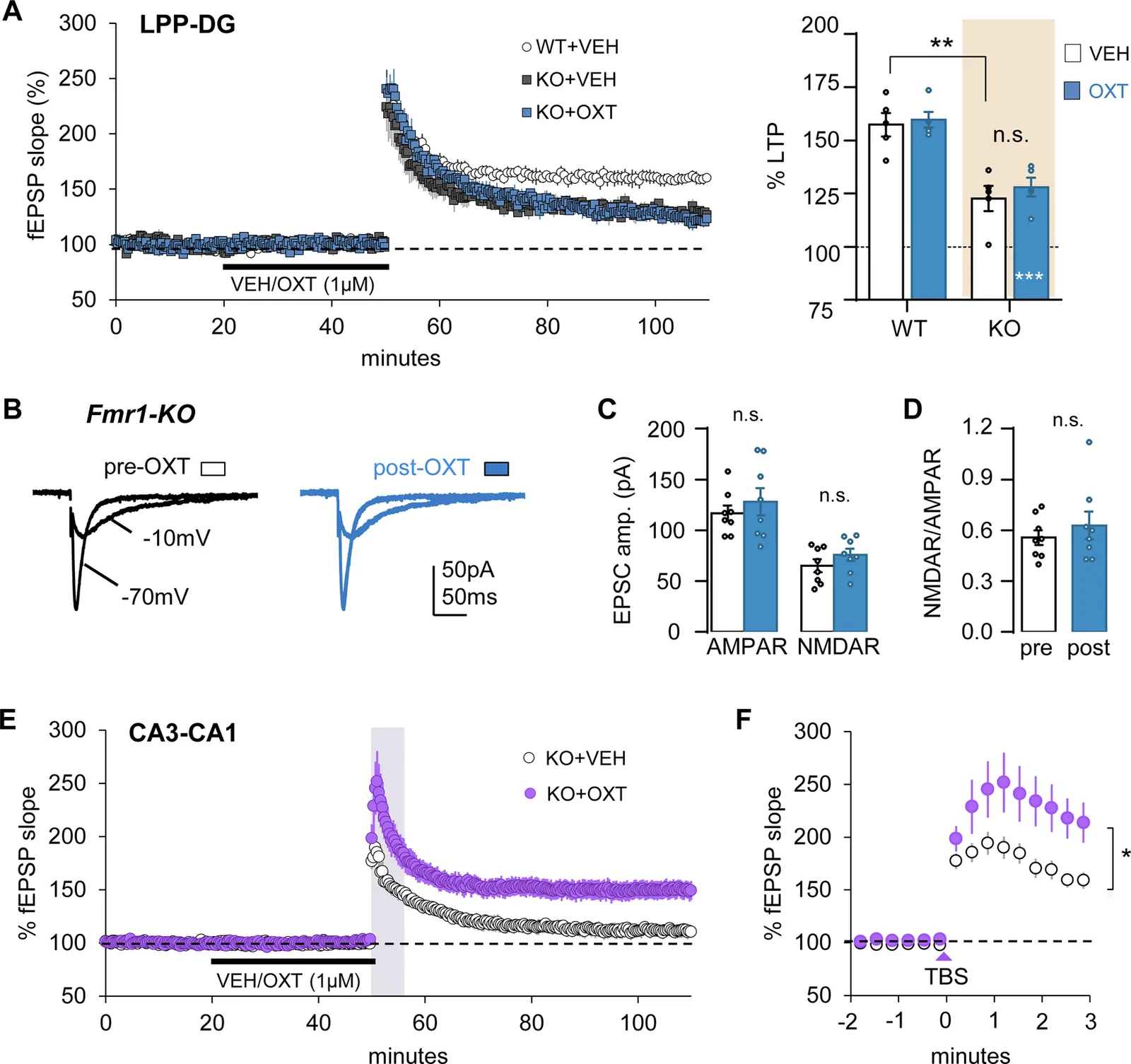
Acute, bath-applied OXT increases S-C LTP in *Fmr1*-KOs but does not affect LPP-LTP. Hippocampal slices from naive adult WT and *Fmr1*-KO mice were treated with vehicle (VEH) or OXT (1 µM) for 30 min before stimulation to induce LTP. **A** *Left*: Plot of LPP fEPSP slopes shows that *Fmr1*-KO slices treated with VEH or OXT have similarly impaired HFS-induced LPP-LTP relative to potentiation in slices from WT mice. *Right:* Bar graph summarizing the percent LTP at 55-60 min post-HFS shows robust LPP-LTP in WT slices, and significantly lower potentiation in KO slices and no effect of OXT infusion (Interaction: *F*_(1,16)_ = 0.093, *p* = 0.76; Genotype: *F*_(1,16)_ = 45.1, *p* < 0.0001; post-hoc Tukey: **p = 0.0017 WT + VEH vs. KO + VEH, n.s. *p* = 0.87 KO + VEH vs. KO + OXT). **B** Whole-cell recordings of *Fmr1*-KO granule cells: EPSCs were elicited by LPP stimulation with cells held at -10mV to assess NMDAR currents or -70mV for AMPAR currents. There was no effect of OXT infusion on **C** LPP AMPAR- (*p* = 0.23, 2-tailed paired t-test) or NMDAR- (*p* = 0.055) EPSCs or **D** the NMDAR/AMPAR current ratio (*p* = 0.35). Legend in (**B**) applies to (**C**, **D**). CA1 field recordings of S-C responses in *Fmr1*-KO slices show that acute OXT infusion (**E**) increased the magnitude of S-C LTP and **F** robustly enhanced the increase in fEPSP slope across the first 3 min post-TBS (plot shows an expansion of the x-axis from 48-53 min in (**E**); Interaction: *F*_(15,180)_ = 5.95, *p* < 0.0001, Treatment: *F*_(1,12)_ = 7.83, *p = 0.016; 2-way RM ANOVA).

Evidence that OXT expression is low in *Fmr1-*KOs and can be increased by postnatal OXT treatment [21, 24] raises the possibility that enduring, treatment-related increases in endogenous OXT efflux normalize plasticity in adult *Fmr1*-KOs. This suggests that acute, bath-applied OXT may reproduce effects of early iOXT on plasticity in *Fmr1*-KO hippocampus. Tests of this possibility produced mixed results. Acute OXT treatment did not influence LPP-LTP in *Fmr1*-KO or WT slices (Fig. 5A). As described, NMDAR-gated LPP EPSCs are abnormally low in *Fmr1*-KO granule cells and normalized by early-life iOXT (Fig. 4G). Nevertheless, acute OXT treatment had no effect on evoked NMDAR-gated responses (Fig. 5B, C) or the NMDAR/AMPAR response ratio (Fig. 5D) in male *Fmr1*-KO slices. Thus, for LPP-DG synapses, acute OXT treatment did not produce the normalization of LPP-LTP or NMDAR operations realized with early-life iOXT administration. In contrast, bath-applied OXT fully restored CA1-LTP elicited by three burst TBS in adult male *Fmr1*-KO slices (Fig. 5E). The early post-TBS growth of fEPSPs otherwise impaired in *Fmr1*-KOs was also normalized by bath-applied OXT (Fig. 5F).

## Discussion

The present studies show that, in addition to enhancing social recognition in *Fmr1*-KOs, the benefits of early postnatal OXT treatment extend to cognitive difficulties that are a salient feature of FXS and other neurodevelopmental disorders [36, 37, 77]. We show that both spatial learning and episodic memory—a form of encoding that is vital for orderly thinking [27, 37, 78, 79]—are severely impaired in adult male *Fmr1*-KOs but fully restored by early-postnatal but not adolescent OXT treatment. Defects in two forms of LTP that are critical for these forms of learning [59, 64] were also corrected by early OXT treatment with effects lasting months into adulthood. Early OXT treatments did not influence behavioral or electrophysiological measures in WT mice. Given that the hippocampus plays a central role in episodic memory [28, 39, 67, 78], it is likely that OXT’s effects on synaptic plasticity are major contributors to improved cognitive function.

Prior studies had shown that early postnatal OXT treatment can restore social recognition in ASD model mice [21, 22, 24, 25]. For *Fmr1*-KOs, this impairment was associated with heightened activity in CA3 during 3-chamber testing and early iOXT reportedly normalized CA3 activity, whereas chemogenetic silencing of CA3 restored social recognition in the mutants [24]. While these findings suggest processes through which early OXT may correct social behavior, they do not offer insight into how similar treatments restore synaptic plasticity or episodic encoding.

Although rodents presumably lack the narrative and autobiographical components of human episodic memory, there is evidence that they utilize an analogous type of encoding [78, 80, 81] that depends on the hippocampus [58]. Specifically, rodents retain information about the identities, locations, and sequence of items encountered during exploration [80, 82–84] and do so without rewards or practice: these are essential features of human episodic memories [85]. Recent work indicates that episodic “What”, “Where”, and “When” encoding depends on distinct neuronal systems: for the tasks employed here, discrete chemogenetic silencing of lateral entorhinal cortex [58], or disturbance of LPP-LTP [59], disrupts acquisition of all three episodic elements whereas silencing medial entorhinal cortex or CA3 leads to discrete loss of “Where” and “When” encoding, respectively [58]. Importantly, we show that male *Fmr1*-KOs exhibit profound deficits in the acquisition of all three major elements of episodic memory, although initial cue sampling times were comparable to those of the wild-types. As in humans, rodent episodes are strongly associated with the context in which they were acquired [86]. Whether this aspect of encoding is affected in *Fmr1*-KOs remains to be tested. Moreover, we have not determined if episodic memory and underlying neurobiological processes are similarly impaired and responsive to iOXT in female *Fmr1*-KOs. Females with FXS are typically heterozygotes for the *Fmr1*-mutation; this, combined with X-inactivation, leads to partial and highly variable reductions in *Fmr1* expression [1]. Nevertheless, females with FXS exhibit impaired executive function and autobiographical memory [2, 87, 88], suggesting there may be deficits in episodic memory amenable to early OXT treatment.

Evidence that early OXT treatment normalized *Fmr1*-KO LTP for *both* the S-C and LPP systems was surprising, given the substantial differences in mechanisms that support plasticity in these two systems. LPP-LTP involves postsynaptic endocannabinoid production and endocannabinoid-dependent increases in neurotransmitter release [75, 76]. In contrast, S-C LTP is induced and expressed post-synaptically, specifically via increases in AMPAR currents [75, 76, 89]. Deficits in LPP-LTP in *Fmr1*-KOs are largely due to impairments in postsynaptic NMDAR currents and retrograde (spine-to-terminal) endocannabinoid signaling [42, 73]. Effects of infused physostigmine described here indicate that early iOXT treatment does not normalize endocannabinoid signaling in the *Fmr1*-KO LPP. However, deficiencies in LPP-evoked NMDAR currents otherwise present in *Fmr1*-KO granule cells were normalized by early iOXT treatment without effects on AMPAR-mediated responses. How OXT elicits this discrete change in NMDAR function is not known. In *Fmr1*-KOs, NMDAR-EPSCs are intact for a second afferent to the granule cell dendrites (i.e., commissural input) [42], suggesting that the defect in LPP synapses reflects an impairment to pathway-specific, presynaptic influences over postsynaptic operations. NMDARs are targeted by multiple kinases and associate with anchoring and signaling proteins [90–93]. They are also regulated by adhesion receptors that stabilize the synapse [94, 95]. Perturbations to these relationships, as described for *Fmr1*-KOs [95], could impair NMDAR operations. The restoration of links between LPP terminals and postsynaptic NMDARs is thus a plausible mechanism through which OXT treatment rescues LPP-LTP that merits further testing.

The above argument does not apply to iOXT-driven recovery of CA1-LTP. There is no evidence for impaired NMDAR function at S-C contacts in *Fmr1*-KOs [43, 73] and, as shown here, responses to LTP-inducing TBS, which reflect NMDAR functions [56, 71], were normal in CA1. There was, however, an effect of genotype on synaptic responses during the first minute post-TBS. The use of threshold induction conditions uncovered what appears to be a previously unrecognized post-TBS phase of S-C fEPSP growth in WTs, and a truncation of this effect in the KOs. It is generally agreed that CA1-LTP is expressed by increases in postsynaptic AMPARs [96] and AMPAR-mediated currents [89, 97, 98], associated with expansion of the activated spine [99, 100] and postsynaptic density [101]. Response facilitation at 20 s post-TBS is eliminated by NMDAR antagonists [86, 102], indicating that response enhancement is due to postsynaptic events even at this early time point. Collectively, these observations suggest that in CA1, the mutation disrupts processes underlying TBS-driven modifications to the postsynaptic compartment, potentially the migration of AMPARs into the newly enlarged active zone. Evidence that early iOXT restored the progressive increase in synaptic responses indicates that in the KOs, heightened OXT exposure during development corrects mechanisms that mediate the initial shift of synapses into their potentiated state.

Acute bath-infusion studies revealed distinct OXT effects on the two axonal systems studied. Although applied OXT had no effect on potentiation in slices from WTs, it fully rescued S-C LTP in *Fmr1*-KOs, including restoration of the initial post-TBS response enhancement (delayed effects of OXT on HFS-induced CA1 LTP have been described [103]). In contrast, bath-applied OXT did not influence LPP-LTP or the associated NMDAR hypofunction in *Fmr1*-KOs. Hypothalamic OXT expression and hippocampal OXT levels are reportedly reduced in *Fmr1*-KOs at P14 [24], and in other ASD models early iOXT increases otherwise deficient hypothalamic OXT expression [21, 104]. These findings, and the present evidence that acute OXT treatment restores S-C LTP in the KOs, suggest that a normalization of endogenous OXT levels may account for the LTP rescue produced by early-life treatment. However, endogenous OXT provided to the hippocampus by hypothalamic afferents would likely be washed out of the slice over the 2 h of aCSF perfusion that preceded physiological testing. An alternative interpretation is that OXT levels in WT mice help maintain cellular (or epigenetic) processes needed to produce stable S-C LTP. If reasonably persistent, such effects would not be dependent upon the presence of OXT within the time frame of the slice experiments. From this perspective, the LTP rescue produced by acute OXT treatment of *Fmr1*-KO slices could indicate that (i) normal OXT-driven events, though persistent, have a rapid onset or (ii) applied OXT engages routes to the potentiated state other than those targeted by endogenous OXT.

The absence of an acute OXT treatment effect on LPP-LTP and LPP NMDAR-EPSCs in male *Fmr1*-KOs argues against the hypothesis that the positive effects of early OXT treatments are due to enhanced adult OXT expression, at least for this particular axonal system. An alternative hypothesis is that the corrective effects of early OXT treatment on LPP-LTP are due to a normalization of developmental trajectories that are essential for adult synaptic plasticity and associated memory functions. This would be consistent with our finding that adolescent OXT treatment had no effect on LPP-LTP or episodic learning in the KOs. The OXT receptor (OXTR) is expressed at relatively high levels in the hippocampus from P7 to P21 [105, 106], ages that overlap the effective treatment period. Thus, early OXT treatment and consequent heightened OXTR functions may compensate for low endogenous OXT levels in the KOs [24] to normalize, during this postnatal period, the emergence of LTP early in the second postnatal week [107, 108]. OXT influences hippocampal dendrites and spines during this period, whereas genetic OXTR deletion changes spine morphology and number [24, 109]. Possibly, the developmental steps that generate the pre- and post-synaptic processes critical for LPP-LTP require OXTR signaling and, having failed to emerge on schedule in the KOs, cannot be reinstated by adolescent or adult OXTR actions. This idea could be tested in studies using genetic or pharmacological manipulations to block OXTR signaling during fixed developmental periods, followed by tests of the integrity of adult LPP-LTP.

Development of the above ideas will require additional information on the mechanistic defects underlying impaired LTP in *Fmr1*-KOs and downstream signaling of the G-protein coupled OXTR [110]. Our prior work on S-C-LTP indicated that the complex machinery responsible for activity-driven spine actin polymerization that is critical for LTP consolidation (e.g., increases in phospho-cofilin and F-actin), is not impaired in male *Fmr1*-KOs [111], whereas TBS-induced activation of Rac GTPase and other proteins that stabilize newly formed actin filaments is disturbed [111, 112]. The OXTR is associated with phospholipase C [103] and engages Rho-GTPases in various cell types [113], and thereby promotes actin network reorganization [113–115]. Whether OXTR signaling influences the postsynaptic actin cytoskeleton and early-life treatment restores mechanisms stabilizing the sub-synaptic cytoskeletal remodeling with CA1 LTP in *Fmr1*-KOs, remains to be determined. Moreover, it is difficult to pose specific hypotheses about the manner in which early OXT treatment restores NMDAR functions at LPP-DG synapses without first explaining how the mutation selectively disrupts receptor operations at this specific site. Clarification of these issues could lead to more specific hypotheses about OXT’s roles in the normal development and later operation of cortical networks.

Overall, the present findings show that OXT treatments limited to the second postnatal week have enduring, normalizing effects on social behavior and the acquisition of hippocampus-dependent spatial and episodic memories in male *Fmr1*-KO mice. These behavioral effects were associated with a restoration of distinct forms of LTP within two axonal systems in *Fmr1*-KO hippocampus [39]. Importantly, the results provide evidence that oxytocin administration during childhood can effectively and enduringly treat cognitive dysfunction in FXS and other autism-associated disorders.

## Supplementary information

SUPPLEMENTARY INFORMATION

## Data Availability

The data that support the findings of this study are available from the corresponding author on reasonable request.
