# Supplementary material for: Lasting effects of early-life oxytocin treatment on LTP and episodic memory in a mouse model of Fragile X syndrome
Source: Neuropsychopharmacology. 2026 Apr 28;51(11):1958–68. doi: 10.1038/s41386-026-02423-0 (PMC13536618; doi:10.1038/s41386-026-02423-0)
Supplement: Supplementary file 1 [file 41386_2026_2423_MOESM1_ESM.docx]

**SUPPLEMENTARY INFORMATION**

**Lasting effects of early-life oxytocin treatment on LTP and episodic memory in a mouse model of Fragile X Syndrome.**

*Chavez* et al.

**Method and Materials.**

***Behavioral Assays.*** Behavioral tests were recorded using an overhead camera; videos were scored by 2 observers blind to group. EthoVision XT (Noldus, Wageningen, Netherlands) was used to collect unbiased, automated measures of locomotion and the total time attending to the two cues during initial exposure sessions. Each mouse was used for at most two paradigms, ≥3 days apart. Behavioral chambers had distinct visual cues on walls. Chambers, objects and scent jars were cleaned with 70% ethanol between uses. Animals were excluded from analyses if they sampled any cue for <1s during the exposure trial. Durations of incidental contact, gnawing, or climbing on the object/cup were excluded from analyses.

The Three-Chamber Sociability Task (1, 2)(Fig. 1B of main text) used a 3-chamber plexiglass arena (63x41-cm floor, 25-cm high) and entailed a habituation period (5 min in empty center chamber, then 10 min exploration of all thee chambers, then a final 5 min in center chamber). For test sessions age-matched FVB male mouse placed under a wire mesh cup was used as the lure. For both recognition and approach phases the time (t) the test mouse sampled a mouse-containing cup or empty cup was scored and expressed using a discrimination index (DI). For social approach, DI=100 x [(t_mouse_ – t_object_)/ t_total_]. For social recognition, DI=100 x [(t_stranger_ – t_familiar_) / t_total_].

Object Location Memory (OLM) (1, 3-5)(Fig. 1E of main text). Mice were habituated to an empty 30x25-cm (21.5-cm heigh) plexiglass arena (5-min/day) for 2 days. The exposure trial entailed 5 min in the arena containing two glass funnels placed in adjacent corners. For testing 24h later, the mouse was allowed 5 min to explore the arena with one funnel displaced toward the arena center. The time the animal spent sampling objects (nose oriented towards and within 0.5-cm of the object) was quantified in seconds. DI=100 x [(t_novel_ − t_familiar_) / t_total_]. Distance traveled was measured using EthoVision XT.

Two-odor Discrimination and Episodic Memory Tasks (6, 7)(Schematics in Figure 2 of main text). These tasks test for discrimination of novelty using odorant cues at concentrations of equal salience for WT and *Fmr1*-KO mice (8). Odorants (**Supplementary** **Table S1**) were dissolved in mineral oil to ~0.1 Pa. and pipetted (~100-120 µL) onto filter papers placed inside glass jars (5.2-cm diameter, 5-cm tall) sealed with perforated lids. Tasks used OLM arenas except the ‘Where’ task which employed a 60x60-cm (30-cm high) plexiglass arena; arena floors had shallow indentions to maintain jar placements across trials. Mice were first habituated to the arena containing unscented jars (5 min). The jars were removed and 5-min later mice were tested in the following paradigms. For the final test trial in each episodic memory task, the mice were exposed to the test cues for 5 minutes, but the DI scores reflected 3-minutes of sampling activity, starting from when the mice first sampled either cue.

Simple 2-Odor Discrimination (6, 7). The mouse was presented with an identical odor pair (E-E) for 3 min. After 21 min delay (to match time from exposure onset to testing in the Serial Odor ‘What’ task), animals were exposed to a new odor pair including familiar odor ‘E’ and novel odor ‘F’ (E-F). DI=100 x [(t_novel_ – t_familiar_)/ t_total_].

Detailed descriptions of the Episodic Serial Odor ‘What’, Serial Odor ‘When’, and ‘Where’ tasks have been described elsewhere (6) and illustrated in main text Figure 2. For the ‘What’ and ‘When’ tasks the DI was calculated as: DI=100 x [(t_older_ – t_recent_)/ t_total_]. For the episodic ‘Where’ task mice were tested 24 hr after exposure to the cues and the DI was calculated as: DI=100 x [(t_sum of switched pair_ – t_sum of stationary pair_)/ t_total_].

***Extracellular Field Electrophysiology.*** Preparation of hippocampal slices for extracellular field recordings for long-term potentiation (LTP) in the CA3-CA1 Schaffer-commissural (S-C) projections and the lateral perforant path (LPP) input to the dentate gyrus (DG) were performed as described (4, 5, 7, 9). For LPP recordings, horizontal 360-µm slices from the third quartile of the septotemporal axis of hippocampus were prepared with a vibratome (VT1000s, Leica Microsystems, Deer Park, IL, USA) and collected into ice-cold, oxygenated, high magnesium- artificial cerebrospinal fluid (aCSF) containing (in mM): 87 NaCl, 26 NaHCO_3_, 25 glucose, 75 sucrose, 2.5 KCl, 1.25 NaH_2_PO_4_, 0.5 CaCl_2_, and 7 MgCl_2_ (320-355 mOsm). This horizontal sectioning preserves the perforant path axons projecting from the entorhinal cortex to the dentate gyrus within the same slice. For CA1 recordings, transverse hippocampal slices from the middle-third section along the septotemporal axis were prepared with a McIllwain chopper (360-µm) and collected into ice-cold aCSF containing (in mM): 124 NaCl, 3 KCl, 1.25 KH_2_PO_4_, 1.5 MgSO_4_, 26 NaHCO_3_, 2.5 CaCl_2_, 10 dextrose. Similarly to the horizontal sectioning for LPP recordings, transverse sectioning preserves the S-C axons across the hippocampal lamellae and maintains its terminating inputs within the same slice. In both S-C and LPP preparations, slices were immediately transferred to an interface recording chamber with continuous aCSF perfusion (1mL/min, 31±1°C). Experiments were initiated ~1.5-2 hrs later. Baseline responses to 0.05Hz stimulation were collected for 20 min before inducing LTP and for 1 hr thereafter.

S-C LTP. The stimulating electrode (twisted, nichrome) was placed in CA1c stratum radiatum (SR), and the recording electrode (glass pipette, filled with 2M NaCl) was placed in CA1b SR, both equidistant from CA1 stratum pyramidale (5). LTP was induced using a three-burst train of theta burst stimulation (TBS: four pulses, 100 Hz per burst; 200 ms between bursts) at baseline stimulation intensity.

LPP-LTP. Stimulating and recording electrodes were placed in the DG outer molecular layer, and LPP stimulation was confirmed by the presence of paired pulse facilitation (40-ms interpulse interval) (10, 11). LTP was induced using a single train of high-frequency stimulation (HFS: 1 second, 100 Hz) at 2x duration and 1.5x intensity of baseline stimulation (7).

Initial slopes and amplitudes of field excitatory postsynaptic potentials (fEPSPs) were digitized at 20kHz using an AC amplifier (A-M Systems, 1700, Sequim, WA) and recorded using NACGather 2.0 (Theta Burst Corp., Irvine, CA). LTP magnitude was assessed by comparing the mean of fEPSP slopes collected 55-60 minutes post-TBS or -HFS to the mean baseline response.

Hippocampal slice bath-treatments. For hippocampal slice field recordings, stock solutions for physostigmine (Tocris, 0622) and OXT (Cell Sciences, CRO300A) were dissolved in water; on the day of use, the stock solution was diluted into aCSF to achieve a final bath concentration of 10 µM and 1 µM, respectively. After collecting 20 min stable recording, the compound was applied to aCSF perfusion line using a syringe pump (6mL/hr; KD Scientific model 100, Holliston, MA). Physostigmine was thus applied to the slice bath for 45 min and OXT or vehicle was applied for 30 min. For OXT treatment experiments, LTP was assessed by comparing mean fEPSP slopes recorded 55–60 min after stimulation relative to the pre-treatment baseline response. For physostigmine treatment experiments, percent decrease in fEPSP slopes was assessed by comparing the baseline with the mean slopes recorded 25–30 min after the onset of the bath-treatment.

***Whole-Cell Current-Clamp.*** Transverse hippocampal slices (350 µm) were generated using a Leica vibrating tissue slicer (VT1000s) into collected into ice-cold, oxygenated, high magnesium aCSF (9, 12). Slices were then transferred to a holding chamber at room temperature and incubated in oxygenated aCSF containing (in mM): 124 NaCl, 3 KCl, 1.25 KH₂PO₄, 1.5 MgSO₄, 26 NaHCO₃, 2.5 CaCl₂, and 10 dextrose (300–310 mOsm, pH 7.4). After recovery for at least 1 hr, slices were placed in a submerged recording chamber continuously perfused (2–3 mL/min; 32°C) with the oxygenated aCSF listed above.

Whole-cell recordings were obtained from DG granule cells with 4–7 MΩ recording pipettes filled with a cesium-based solution containing (in mM): 140 CsMeSO₄, 8 CsCl, 10 HEPES, 0.2 EGTA, 2 QX-314, 2 Mg-ATP, and 0.3 Na-GTP, adjusted to 290–295 mOsm and pH 7.25 (13). The stimulating electrode, in the DG outer molecular layer, was used to evoke LPP-specific responses. Paired-pulse stimulation (40-ms interpulse interval) was applied to verify facilitation characteristic of the LPP–DG synapse. Excitatory postsynaptic currents (EPSCs) were recorded by clamping the cell at -70 mV in the presence of 50-µM picrotoxin (9). NMDAR- to AMPA receptor (AMPAR) -mediated EPSC ratios were calculated as the ratio of current at 30-ms post-stimulus onset with -10 mV holding potential to peak current at -70 mV (12). Recordings were analyzed using Clampfit 10.6 (Molecular Devices, San Jose, CA).

*Each odorant was dissolved in mineral oil (~0.1 Pa).

**Supplementary Table 1.** List of odorants used in behavioral tasks

| **Odorant ID** | **Odorant (name, vendor)** | **Concentration (odorant: mineral oil)** |
| --- | --- | --- |
| A | (+) -Limonene (>97% purity, *Sigma- Aldrich*) | 1:4000 |
| B | Cyclohexyl ethyl acetate (>97%, *International Flavors & Fragrances Inc.*) | 1.97:4000 |
| C | Citronellal 96% (*Alfa Aesar*) | 1.5:4000 |
| D | Octyl aldehyde 99% (*Acros Organics*) | 1.5:4000 |
| E | Anisole 99% (*Acros Organics*) | 0.85:4000 |
| F | 1-Pentanol 99% (*Acros Organics*) | 1.36:4000 |

| **Supplementary Table 2.** Statistical values for behavioral analyses.  **Treatment P7-P13** | | | | | | | | |
| --- | --- | --- | --- | --- | --- | --- | --- | --- |
| Figure | Test | |  | | Mean ± SEM | | | Two-way ANOVA |
| 1C | Social Approach | object vs **S1** | | WT-SAL: 49.2±5.8  WT-OXT: 56.1±4.3  KO-SAL: 55.3±5.9  KO-OXT: 67.7±4.9 | | Interaction: F_(1,40)_=0.25, p=0.62  Genotype: F_(1,40)_=2.68, p=0.11  Treatment: F_(1,40)_=3.16, p=0.08 | | |
|  |  |  | |  | | Post hoc Tukey | | |
|  |  |  | |  | | WT-SAL vs. WT-OXT: p=0.78  WT-SAL vs. KO-SAL: p=0.83  WT-SAL vs. KO-OXT: p=0.09  WT-OXT vs. KO-SAL: p=0.99  WT-OXT vs. KO-OXT: p=0.48  KO-SAL vs. KO-OXT: p=0.42 | | |
| 1D | Social Recognition | S1 vs **S2** | | WT-SAL: 28.9±7.8  WT-OXT: 25.1±6.8  KO-SAL: -0.97±7.8  KO-OXT: 28.1±4.1 | | Interaction: F_(1,37)_=5.15, p=0.03  Genotype: F_(1,37)_=3.46, p=0.07  Treatment: F_(1,37)_=3.0, p=0.09 | | |
|  |  |  | |  | | Post hoc Tukey | | |
|  |  |  | |  | | WT-SAL vs. WT-OXT: p=0.98  WT-SAL vs. KO-SAL: *p=0.02  WT-SAL vs. KO-OXT: p=0.99  WT-OXT vs. KO-SAL: p=0.054  WT-OXT vs. KO-OXT: p=0.99  KO-SAL vs. KO-OXT: *p=0.047 | | |
| 1F | OLM | stationary vs **moved** | | WT-SAL: 25.4±5.1  WT-OXT: 20.2±6.6  KO-SAL: -2.2±2.4  KO-OXT: 26.6±4.3 | | Interaction: F_(1,40)_=11.63, p=0.002  Genotype: F_(1,40)_=4.48, p=0.04  Treatment: F_(1,40)_=5.57, p=0.02 | | |
|  |  |  | |  | | Post hoc Tukey | | |
|  |  |  | |  | | WT-SAL vs. WT-OXT: p=0.87  WT-SAL vs. KO-SAL:** p=0.002  WT-SAL vs. KO-OXT: p=0.99  WT-OXT vs. KO-SAL: *p=0.012  WT-OXT vs. KO-OXT: p=0.80  KO-SAL vs. KO-OXT: **p=0.0015 | | |
| 1G | Distance Travelled | Empty | | WT-SAL:17.1±0.80  WT-OXT:15.0±0.63  KO-SAL: 20.4±0.9  KO-OXT: 18.4±0.68 | | Interaction: F_(1,42)_=0.0081, p=0.93  Genotype: F_(1,42)_=18.8, p<0.0001  Treatment: F_(1,42)_=7.17, p=0.011 | | |
|  |  |  | |  | | Post hoc Tukey | | |
|  |  |  | |  | | WT-SAL vs. WT-OXT: p=0.20  WT-SAL vs. KO-SAL: *p=0.19  WT-SAL vs. KO-OXT: p=0.66  WT-OXT vs. KO-SAL: ****p<0.0001  WT-OXT vs. KO-OXT: *p=0.020  KO-SAL vs. KO-OXT: p=0.29 | | |
|  |  | Exposure | | WT-SAL: 22.1±1.9  WT-OXT: 19.2±1.6  KO-SAL: 24.8±2.4  KO-OXT: 24.5±1.1 | | Interaction: F_(1,42)_=0.52, p=0.48  Genotype: F_(1,42)_=4.64, p=0.04  Treatment: F_(1,42)_=0.76, p=0.39 | | |
|  |  |  | |  | | Post hoc Tukey | | |
|  |  |  | |  | | WT-SAL vs. WT-OXT: p=0.66  WT-SAL vs. KO-SAL: p=0.73  WT-SAL vs. KO-OXT: p=0.81  WT-OXT vs. KO-SAL: p=0.14  WT-OXT vs. KO-OXT: p=0.21  KO-SAL vs. KO-OXT: p=0.99 | | |
|  |  | Test | | WT-SAL: 17.2±1.6  WT-OXT: 15.5±1.3  KO-SAL: 23.2±1.7  KO-OXT: 22.4±1.4 | | Interaction: F_(1,42)_=0.08, p=0.77  Genotype: F_(1,42)_=17.5, p=0.0001  Treatment: F_(1,42)_=0.64, p=0.43 | | |
|  |  |  | |  | | Post hoc Tukey | | |
|  |  |  | |  | | WT-SAL vs. WT-OXT: p=0.86  WT-SAL vs. KO-SAL: *p=0.035  WT-SAL vs. KO-OXT: p=0.11  WT-OXT vs. KO-SAL: **p=0.0043  WT-OXT vs. KO-OXT: *p=0.018  KO-SAL vs. KO-OXT: p= | | |
| 2B | 2-odor | familiar vs **novel** | | WT-SAL: 39.6±4.6  WT-OXT: 42.5±4.2  KO-SAL: 43.7±5.6  KO-OXT: 34.4±4.2 | | Interaction: F_(1,43)_=1.72, p=0.20  Genotype: F_(1,43)_=0.18, p=0.68  Treatment: F_(1,43)_=0.48, p=0.49 | | |
|  |  |  | |  | | Post hoc Tukey | | |
|  |  |  | |  | | WT-SAL vs. WT-OXT: p=0.97  WT-SAL vs. KO-SAL: p=0.92  WT-SAL vs. KO-OXT: p=0.86  WT-OXT vs. KO-SAL: p=0.99  WT-OXT vs. KO-OXT: p=0.61  KO-SAL vs. KO-OXT: p=0.52 | | |
| 2D | What | familiar vs **novel** | | WT-SAL: 37.0±5.1  WT-OXT: 35.1±7.6  KO-SAL: -8.0±6.8  KO-OXT: 31.7±5.3 | | Interaction: F_(1,50)_=10.8, p=0.002  Genotype: F_(1,50)_=14.6, p=0.0004  Treatment: F_(1,50)_=8.91, p=0.004 | | |
|  |  |  | |  | | Post hoc Tukey | | |
|  |  |  | |  | | WT-SAL vs. WT-OXT: p=0.99  WT-SAL vs. KO-SAL:****p<0.0001  WT-SAL vs. KO-OXT: p=0.93  WT-OXT vs. KO-SAL:****p<0.0001  WT-OXT vs. KO-OXT: p=0.98  KO-SAL vs. KO-OXT: ***p=0.002 | | |
| 2F | When | recent vs **older** *(odor B vs C) | | WT-SAL: 35.4±5.9  WT-OXT: 43.4±5.6  KO-SAL: -2.2±10.3  KO-OXT: 48.9±7.5 | | Interaction: F_(1,35)_=7.34, p=0.01  Genotype: F_(1,35)_=4.1, p=0.05  Treatment: F_(1,35)_=13.9, p=0.0007 | | |
|  |  |  | |  | | Post hoc Tukey | | |
|  |  |  | |  | | WT-SAL vs. WT-OXT: p=0.90  WT-SAL vs. KO-SAL: **p=0.0092  WT-SAL vs. KO-OXT: p=0.64  WT-OXT vs. KO-SAL: **p=0.0013  WT-OXT vs. KO-OXT: p=0.96  KO-SAL vs. KO-OXT: ***p=0.0002 | | |
| 2H | Where | stationary vs **switched** | | WT-SAL: 50.0±7.9  WT-OXT: 57.8±6.1  KO-SAL: -10.6±7.1  KO-OXT: 43.0±6.1 | | Interaction: F_(1,29)_=11.0, p=0.003  Genotype: F_(1,29)_=29.8, p<0.0001  Treatment: F_(1,29)_=19.8, p=0.0001 | | |
|  |  |  | |  | | Post hoc Tukey | | |
|  |  |  | |  | | WT-SAL vs. WT-OXT: p=0.87  WT-SAL vs. KO-SAL: ****p<0.0001  WT-SAL vs. KO-OXT: p=0.88  WT-OXT vs. KO-SAL: ****p<0.0001  WT-OXT vs. KO-OXT: p=0.46  KO-SAL vs. KO-OXT: ****p<0.0001 | | |
| Treatment P30-P36 | | | | | | | | |
| S6B | 2-odor | familiar vs **novel** | | WT-SAL: 48.3±4.5  WT-OXT: 44.0±6.1  KO-SAL: 39.2±5.4  KO-OXT: 46.4±6.9 | | Interaction: F_(1,43)_=0.93, p=0.34  Genotype: F_(1,43)_=0.32, p=0.58  Treatment: F_(1,43)_=0.06, p=0.82 | | |
|  |  |  | |  | | Post hoc Tukey | | |
|  |  |  | |  | | WT-SAL vs. WT-OXT: p=0.96  WT-SAL vs. KO-SAL: p=0.72  WT-SAL vs. KO-OXT: p=0.99  WT-OXT vs. KO-SAL: p=0.94  WT-OXT vs. KO-OXT: p=0.99  KO-SAL vs. KO-OXT: p=0.83 | | |
| 4I | What | familiar vs **novel** | | WT-SAL: 31.2±6.8  WT-OXT: 23.2±4.1  KO-SAL: -5.6±5.6  KO-OXT: -13.0±6.0 | | | Interaction: F_(1,50)_=0.003, p=0.96  Genotype: F_(1,50)_=40.0, p<0.0001  Treatment: F_(1,50)_=1.80, p=0.19 | |
|  |  |  | |  | | | Post hoc Tukey | |
|  |  |  | |  | | | WT-SAL vs. WT-OXT: p=0.78  WT-SAL vs. KO-SAL: ***p=0.0002  WT-SAL vs. KO-OXT: ****p<0.0001  WT-OXT vs. KO-SAL: **p=0.0051  WT-OXT vs. KO-OXT: ***p=0.0004  KO-SAL vs. KO-OXT: p=0.78 | |

**Bolded text**: preferred object or odor.

| **Treatment P7-P13**  **Supplementary Table 3.** Statistical values for raw supplementary data behavioral analyses. | | | | | | | | |
| --- | --- | --- | --- | --- | --- | --- | --- | --- |
| Figure | Test |  | | Paired t-test or F-value (ANOVA) | | | |  |
| S1A | Social Approach | object vs **S1** | | WT-SAL  WT-OXT  KO-SAL  KO-OXT | | t_11_=6.606, p<0.0001  t_10_=7.724, p<0.0001  t_10_=4.193, p=0.0018  t_8_=9.692, p<0.0001 | |  |
| S1B | Social Recognition | S1 vs **S2** | | WT-SAL  WT-OXT  KO-SAL  KO-OXT | | t_11_=3.225, p=0.0079  t_10_=3.355, p=0.0073  t_10_=0.6457, p=0.5330  t_7_=2.711, p=0.0302 | |  |
| S1C | OLM | stationary vs **moved** | | WT-SAL  WT-OXT  KO-SAL  KO-OXT | | t_10_=4.854, p=0.0007  t_11_=2.895, p=0.0146  t_10_=0.8717, p=0.4038  t_9_=2.417, p=0.0388 | |  |
| S2A | 2-odor | Sampling time | | Interaction  Treatment  Genotype | | F_(1, 42)_=1.046, p=0.3124  F_(1, 42)_=0.02193, p=0.8830  F_(1, 42)_=0.4805, p=0.4920 | |  |
| S2B | What | Sampling time | | Interaction  Treatment  Genotype | | F_(1, 51)_=0.0907, p=0.765  F_(1, 51)_=3.2924, p=0.075  F_(1, 51)_=0.9494, p=0.334 | |  |
| S2C | When | Sampling time | | Interaction  Treatment  Genotype | | F_(1, 36)_=0.9395, p=0.339  F_(1, 36)_=0.9074, p=0.347  F_(1, 36)_=0.0234, p=0.879 | |  |
| S2D | Where | Sampling time | | Interaction  Treatment  Genotype | | F_(1, 29)_=0.08126, p=0.7776  F_(1, 29)_=0.1015, p=0.7523  F_(1, 29)_=1.890, p=0.1797 | |  |
| S3A | 2-odor | familiar vs **novel** | | WT-SAL  WT-OXT  KO-SAL  KO-OXT | | t_11_=6.287, p<0.0001  t_12_=6.368, p<0.0001  t_10_=5.736, p=0.0002  t_10_=6.128, p=0.0001 | |  |
| S3B | What | familiar vs **novel** | | WT-SAL  WT-OXT  KO-SAL  KO-OXT | | t_13_=4.412, p=0.0007  t_10_=3.027, p=0.0127  t_15_=1.801, p=0.0919  t_12_=3.901, p=0.0021 | |  |
| S3C | When | recent vs **older** *(odor B vs odor C) | | WT-SAL  WT-OXT  KO-SAL  KO-OXT | | t_8_=2.762, p=0.0246  t_8_=5.468, p=0.0006  t_11_=0.3355, p=0.7676  t_8_=3.654, p=0.0065 | |  |
| S3D | Where | stationary vs **switched** | | WT-SAL  WT-OXT  KO-SAL  KO-OXT | | t_7_=3.221, p=0.0146  t_6_=6.706, p=0.0005  t_8_=0.8705, p=0.4094  t_8_=4.924, p=0.0012 | |  |
| S4A | 2-odor | Distance Travelled | | Interaction  Treatment  Genotype | | F_(1, 42)_=1.046, p=0.3124  F_(1, 42)_=0.02193, p=0.8830  F_(1, 42)_=0.4805, p=0.4920 | |  |
| S4B | What | Distance Travelled | | Interaction  Treatment  Genotype | | F_(1, 51)_=0.0621, p=0.804  F_(1, 51)_=5.2, p=0.027  F_(1, 51)_=10.3807, p=0.002 | |  |
| S4C | When | Distance Travelled | | Interaction  Treatment  Genotype | | F_(1, 36)_=0.269, p=0.607  F_(1, 36)_=7.383, p=0.01  F_(1, 36)_=10.111, p=0.003 | |  |
| S4D | Where | Distance Travelled | | Interaction  Treatment  Genotype | | F_(1, 29)_=0.0203, p=0.888  F_(1, 29)_=2.0186, p=0.166  F_(1, 29)_=18.3025, p<0.001 | |  |
| **Treatment P30-P36** | | | | | | | |  |
| S6C | 2-odor | | familiar vs **novel**  Sampling Time | | WT-SAL  WT-OXT  KO-SAL  KO-OXT | | t_10_=8.792, p<0.0001  t_11_=5.921, p=0.0001  t_10_=5.863, p=0.0002  t_12_=4.543, p=0.0007 |  |
| S6D | What | | familiar vs **novel**  Sampling Time | | WT-SAL  WT-OXT  KO-SAL  KO-OXT | | t_12_=3.121, p=0.0088  t_11_=4.701, p=0.0006  t_15_=0.4782, p=0.6399  t_13_=1.208, p=0.2487 |  |

**Bolded text**: WT preferentially explored object or odor. Within group comparisons: paired t-test (two-tailed)

**Supplementary Figure 1**

**Supplementary Figure 1: Sampling times during the test trial for three-chamber sociability task and object location memory (OLM) paradigms.** (**A, B**) Graphs show mean raw sampling time (seconds) used to calculate the discrimination indices for the three-chamber task during the Approach (see Fig. 1C) and Recognition (see Fig. 1D) phases. (**A**) *Approach:* Mean sampling time of the stranger mouse (S1) is greater than that of the object for all groups. (**B**) *Recognition*: Both WT groups (WT-SAL and WT-OXT) and KOs given OXT (KO-OXT) exhibited greater sampling times for the novel stranger mouse (S2) vs. the familiar mouse (S1). However, the KO-SAL group did not show a bias. (**C**) Graphs show the mean raw sampling time for the OLM testing trial (see Fig. 1F). Time spent sampling the moved object vs. the stationary object was greater in both WT groups and OXT-treated KOs, whereas KO-SAL mice exhibited no preference for the moved object. *Statistics*: 2-tailed paired t-test: n.s. p>0.05, *p<0.05, **p<0.01, ***p<0.001, ****p<0.0001. Detailed statistical analyses are provided in Supplementary Table 3. Data presented as mean ± SEM.

**Supplementary Figure 2**

**Supplementary Figure 2: Sampling times during the initial cue exposure trials for episodic memory task paradigms.** (**A-D**) Mean time (seconds) spent on cues in the initial exposure trials prior to the test phase presented in Fig. 2B (2-odor), Fig. 2D (‘What’), Fig. 2F (‘When’), and Fig. 2H (‘Where’). Sampling times were comparable across groups, with no effects of genotype or treatment in the 2-odor discrimination task (**A**), ‘What’ task (**B**), ‘When’ task (**C**), and ‘Where’ task (**D**). *Statistics*: repeated-measures mixed ANOVA for panels B and C; two-way ANOVA for panels A and D. Detailed statistical analyses are provided in Supplementary Table 3. Data presented as mean ± SEM.

**Supplementary Figure 3**

**Supplementary Figure 3: Sampling times during the test trial for 2-Odor discrimination and episodic memory tasks.** (**A-D**) Graphs show the sampling times (seconds) of test odors used to calculate each animal’s discrimination index presented in Fig. 2B (2-Odor), Fig. 2D (‘What’), Fig. 2F (‘When’), and Fig. 2H (‘Where’). (**A**) *2-Odor:* All groups exhibited greater sampling times for the novel odor vs. the familiar odor. (**B**) *‘What’ task*: KOs given SAL (KO-SAL) had similar sampling times for the novel and familiar odors, whereas the other three groups sampled the novel odor more. (**C**) *‘When’ task*: Both WT groups and KOs given OXT (KO-OXT) spent more time sampling the earlier (Older) presented odor vs. the later (Recent) presented odor, whereas the KO-SAL group sampled both odors similarly. (**D**) *‘Where’ task*: Sampling times for the KO-SAL group were comparable between the switched and stationary odor pairs, while both WT groups and the KO-OXT group sampled the switched pair for a greater amount of time. *Statistics*: 2-tailed paired t-test: n.s. p>0.05, *p<0.05, **p<0.01, ***p<0.001, ****p<0.0001. Detailed statistical analyses are provided in Supplementary Table 3. Data presented as mean ± SEM.

**Supplementary Figure 4**

**Supplementary Figure 4: Distance travelled for episodic memory task paradigms.** (**A-D**) Mean distance travelled in centimeters during the initial cue exposure trials and testing trial for the 2-odor discrimination task (**A**), ‘What’ (**B**), ‘When’ (**C**), and Where task (**D**). Across all tasks, *Fmr1*-KO mice travelled significantly farther than wild-type mice (genotype effect, p<0.007), whereas treatment had no effect (p>0.05). *Statistics*: repeated-measures mixed ANOVA for all panels; detailed statistical results are provided in Supplementary Table 3. Data are presented as mean ± SEM.

**Supplementary Figure 5**

**Supplementary Figure 5: Representative traces for CA3-CA1 and LPP-DG before and after LTP stimulation.** OXT and Sal treatments were given to WTs and *Fmr1*-KOs at either P7-13 (**A,B**) or P30-36 (**C**) and recordings were collected from hippocampal slices derived from adult animals. (**A**) Representative traces for S-C CA1 LTP show pre-TBS (black) and 55-60 min post-TBS fEPSP responses (purple) for each group. (**B,C**) Representative traces for LPP-DG LTP show pre-HFS (black) and 55-60 min post-HFS fEPSP responses for P7-13 treatments (B, blue) and P30-36 treatments (C, orange) for each group. Scale bars are 1mV, 10ms for all panels.

**Supplementary Figure 6**

**Supplementary Figure 6: Adolescence OXT treatment and effects on 2-odor discrimination and episodic ‘What” task.** (**A**) Schematic showing the adolescence treatment paradigm. WT and *Fmr1*-KO mice received daily intranasal OXT or SAL treatment from P30 to P36. Effects of treatment were assessed at 2-4 months of age. (**B**) In the 2-odor discrimination task, all groups successfully discriminated the novel odor in the test session (Interaction: F_(1,45)_= 0.643, p=0.427). (**C,D**) Sampling times (seconds) during the test trial used to calculate the discrimination indices presented in Fig. S6B (2-odor) and Fig. 4I (‘What’) for P30-P36 treated mice. (**C**) *2-Odor*: All four groups exhibited greater sampling times for the novel odor vs. the familiar odor. (**D**) *‘What’ task:* Both WT groups sampled the novel odor for greater time as compared to the familiar odor. By contrast, both the SAL-treated and OXT-treated KO groups sampled the two odors for similar amounts of time. *Statistics:* 2-way ANOVA for panel B. 2-tailed paired t-test for panel C and D*.* n.s. p>0.05, **p<0.01, ***p<0.001, ****p<0.0001. Detailed statistical analyses are provided in Supplementary Table 2 for panel B, and Supplementary Table 3 for panels C and D. Data presented as mean ± SEM.

**Supplementary Material References**

1.	Seese RR, Le AA, Wang K, Cox CD, Lynch G, Gall CM (2020): A TrkB agonist and ampakine rescue synaptic plasticity and multiple forms of memory in a mouse model of intellectual disability. *Neurobiol Dis*. 134:104604.

2.	Moy SS, Nadler JJ, Perez A, Barbaro RP, Johns JM, Magnuson TR, et al. (2004): Sociability and preference for social novelty in five inbred strains: an approach to assess autistic-like behavior in mice. *Genes Brain Behav*. 3:287-302.

3.	Seese RR, Wang K, Yao YQ, Lynch G, Gall CM (2014): Spaced training rescues memory and ERK1/2 signaling in fragile X syndrome model mice. *Proc Natl Acad Sci U S A*. 111:16907-16912.

4.	Le AA, Lauterborn JC, Jia Y, Wang W, Cox CD, Gall CM, et al. (2022): Prepubescent female rodents have enhanced hippocampal LTP and learning relative to males, reversing in adulthood as inhibition increases. *Nat Neurosci*. 25:180-190.

5.	Wang W, Le AA, Hou B, Lauterborn JC, Cox CD, Levin ER, et al. (2018): Memory-related synaptic plasticity is sexually dimorphic in rodent hippocampus. *J Neurosci*. 38:7935-7951.

6.	Cox BM, Cox CD, Gunn BG, Le AA, Inshishian VC, Gall CM, et al. (2019): Acquisition of temporal order requires an intact CA3 commissural/associational (C/A) feedback system in mice. *Commun Biol*. 2:251.

7.	Wang W, Cox BM, Jia Y, Le AA, Cox CD, Jung KM, et al. (2018): Treating a novel plasticity defect rescues episodic memory in Fragile X model mice. *Mol Psychiatry*. 23:1798-1806.

8.	Schilit Nitenson A, Stackpole EE, Truszkowski TL, Midroit M, Fallon JR, Bath KG (2015): Fragile X mental retardation protein regulates olfactory sensitivity but not odorant discrimination. *Chem Senses*. 40:345-350.

9.	Quintanilla J, Jia Y, Lauterborn JC, Pruess BS, Le AA, Cox CD, et al. (2022): Novel types of frequency filtering in the lateral perforant path projections to dentate gyrus. *J Physiol*. 600:3865-3896.

10.	McNaughton BL, Barnes CA (1977): Physiological identification and analysis of dentate granule cell responses to stimulation of the medial and lateral perforant pathways in the rat. *J Comp Neurol*. 175:439-454.

11.	Christie BR, Abraham WC (1994): Differential regulation of paired-pulse plasticity following LTP in the dentate gyrus. *Neuroreport*. 5:385-388.

12.	Wang W, Jia Y, Pham DT, Palmer LC, Jung KM, Cox CD, et al. (2018): Atypical Endocannabinoid Signaling Initiates a New Form of Memory-Related Plasticity at a Cortical Input to Hippocampus. *Cereb Cortex*. 28:2253-2266.

13.	Trieu BH, Kramar EA, Cox CD, Jia Y, Wang W, Gall CM, et al. (2015): Pronounced differences in signal processing and synaptic plasticity between piriform-hippocampal network stages: a prominent role for adenosine. *J Physiol*. 593:2889-2907.
